# To: Posterior reversible encephalopathy syndrome in a child with severe multisystem inflammatory syndrome due to COVID-19

**DOI:** 10.5935/2965-2774.20230283-en

**Published:** 2023

**Authors:** Carla Alessandra Scorza, Ana Claudia Fiorini, Fulvio Alexandre Scorza, Josef Finsterer

**Affiliations:** 1 Discipline of Neuroscience, Escola Paulista de Medicina, Universidade Federal de São Paulo - São Paulo (SP), Brazil; 2 Department of Scpeech Therapy, Escola Paulista de Medicina, Universidade Federal de São Paulo - São Paulo (SP), Brazil; 3 Neurology and Neurophysiology Center - Vienna, Austria

## To the editor

We read with interest the article by Dominguez-Rojas et al. about a severe acute respiratory syndrome coronavirus 2 (SARS-CoV-2) polymerase chain reaction (PCR)-negative 9-year-old male who underwent laparotomy for suspected acute abdomen (vomiting, abdominal pain, diarrhea), which was noninformative.^(1)^ On postoperative day one, the patient experienced respiratory insufficiency attributed to pneumonia with pleural effusion requiring mechanical ventilation and noradrenergic support.^(1)^ Although weaning was feasible 15 days after intubation, the patient deteriorated again, manifesting with bilateral plantar fasciitis, delusions, suicidal ideation, psychomotor agitation, and two generalized seizures.^(1)^ Cerebral magnetic resonance imaging (MRI) revealed bilateral T2 hyperintensities in the white matter of the occipital lobes, leading to a diagnosis of posterior reversible encephalopathy syndrome (PRES) due to multisystem inflammatory syndrome in childhood; the patient was successfully treated with intravenous immunoglobulins, resulting in almost complete recovery at the three-week follow-up after discharge.^(1)^ The study is appealing but raises concerns that should be discussed.

We disagree with the diagnosis of PRES. The PRES is usually associated with arterial hypertension.^(2)^ However, the patient had no history of arterial hypertension and either arterial hypotension or normal blood pressure values during hospitalization in the intensive care unit.^(1)^ Were elevated blood pressure values ever measured? Although PRES can also develop in the absence of arterial hypertension,^(3)^ this is rather rare. Differential diagnoses that should have been ruled out include cerebral hypoxia (the patient experienced hypoxia prior to intubation), acute disseminated encephalomyelitis (ADEM), immune encephalitis, and venous sinus thrombosis. A shortcoming in this respect is that the patient did not undergo investigations of the cerebrospinal fluid. Cerebrospinal fluid investigations are necessary to particularly rule out ADEM and encephalitis.

We also disagree with the diagnosis of COVID-19.^(1)^ The patient never tested positive for SARS-CoV-2 RNA by PCR.^(1)^ Elevation of neutralizing IgG antibodies does not necessarily indicate an acute infection, as elevation of anti-SARS-CoV-2 IgG antibodies starts approximately 14 days after contamination and persists for up to several months.^(4)^

Furthermore, we disagree that T2/FLAIR hyperintensities are indicative of vasogenic edema.^(1)^ Vasogenic edema on multimodal MRI is characterized by diffusion-weighted imaging and apparent diffusion coefficient map hyperintensities.

The reference limits of the parameter proBNP were given as > 1pg/mL in table 1.^(1)^ Accordingly, the measured value of 282pg/mL is normal.^(1)^ However, the values were assessed as “high” and described as elevated in the main body of the text.^(1)^ This discrepancy should be solved. We should be told if proBNP was indeed normal or increased. Because the patient was diagnosed with heart failure and had reduced systolic function on echocardiography, it is conceivable that proBNP was elevated.

Overall, this interesting study has limitations that call the results and their interpretation into question. Clarifying these weaknesses would strengthen the conclusions and could improve the study. Diagnosing SARS-CoV-2-related PRES requires diagnosing COVID-19 and PRES according to established criteria.
